# Factors influencing the preference for purchasing generic drugs in a Southern Brazilian city

**DOI:** 10.1590/S1518-8787.2017051006786

**Published:** 2017-06-20

**Authors:** Marília Cruz Guttier, Marysabel Pinto Telis Silveira, Vera Lucia Luiza, Andréa Dâmaso Bertoldi

**Affiliations:** I Curso de Farmácia. Centro de Ciências Químicas, Farmacêuticas e de Alimentos. Universidade Federal de Pelotas. Pelotas, RS, Brasil; II Departamento de Fisiologia e Farmacologia, Instituto de Biologia. Universidade Federal de Pelotas. Pelotas, RS, Brasil; III Escola Nacional de Saúde Pública Sérgio Arouca. Fundação Oswaldo Cruz. Rio de Janeiro, RJ, Brasil; IV Programa de Pós-Graduação em Epidemiologia. Faculdade de Medicina. Universidade Federal de Pelotas. Pelotas, RS, Brasil

**Keywords:** Drugs, Generic, economics, Patient Medication Knowledge, Consumer Behavior, Socioeconomic Factors, Cross-Sectional Studies

## Abstract

**OBJECTIVE:**

The objective of this study is to identify factors associated with the preference for purchasing generic drugs in a medium-sized municipality in Southern Brazil.

**METHODS:**

We have analyzed data from a population-based cross-sectional study conducted in 2012 with a sample of 2,856 adults (≥ 20 years old). The preference for purchasing generic drugs was the main outcome. The explanatory variables were the demographic and socioeconomic variables. Statistical analyses included Poisson regressions.

**RESULTS:**

The preference for purchasing generic drugs was 63.2% (95%CI 61.4–64.9). The variables correlated with this preference in the fully adjusted models were: male (prevalence ratio [PR] = 1.08; 95%CI 1.03–1.14), age of 20–39 years (PR = 1.10; 95%CI 1.02–1.20), low socioeconomic status (PR = 1.15; 95%CI 1.03–1.28), and good knowledge about generic drugs (PR= 4.66; 95%CI 2.89–7.52). Among those who preferred to purchase generic drugs, 55.1% have reported accepting to replace the prescribed drug (if not a generic) with the equivalent generic drug. Another correlate of the preference for purchasing generic drugs was because individuals consider their quality equivalent to reference medicines (PR = 2.15; 95%CI 1.93–2.41).

**CONCLUSIONS:**

Knowledge about generic drugs was the main correlate of the preference for purchasing generic drugs. The greater the knowledge or positive perception about generic drugs, the greater is the preference to purchase them. Therefore, educational campaigns for healthcare professionals and consumers appear to be the best strategy for expanding the use of generic drugs in Brazil.

## INTRODUCTION

The challenges for expanding access to medicines and reducing expenditures on these products have resulted in major changes in the Brazilian pharmaceutical sector. The establishment of policies such as the National Drug Policy^25^ and the National Policy of Pharmaceutical Assistance^26^ are initiatives to ensure access to medicines and to promote their rational use.

The Generic Drug Law implemented in Brazil in 1999^25^ also aims to facilitate access of persons to drug treatment, offering medicines with assured quality and low prices, reducing household expenditure on them^2^.

Since the implementation of the Generic Drug Law^25^, three types of drugs are being marketed in Brazil: reference drugs (innovative drugs, brands), generic drugs (drugs designated by the Brazilian Nonproprietary Name [DCB], usually produced after the expiration of patent protection, which must be interchangeable with the reference drug), and similar medicines (not interchangeable with the reference drug and marketed under a brand name)^25^.

Most studies on generic drugs have focused on the knowledge, beliefs, and perceptions of pharmacists, physicians, and patients about these drugs^10,11^. We have relatively good knowledge on users of generic drugs in Brazil^8,27,30^ and abroad^20,21^, but the literature of upper middle income countries lacks studies on the factors that influence the purchasing decision of consumers between generic and brand drugs^14,24,32^.

In Brazil, according to the Brazilian Association of Generic Drug Industries (*Pro Genericos*)^a^, generic drugs reached 28% of the sales volume in units sold and 24.8% of the total financial value, considering data from the Intercontinental Marketing Services (IMS) Health, in 2014. Studies on the prevalence of the use of generic drugs have found values ranging from 3.6% to 45%^8,9,27,35^. It is worth noting that studies carried out earlier than 2007 found lower prevalence of use of generic drugs^8,9,35^ when compared to a national study conducted in 2013^27^.

Studies from Italy^12^ and Portugal^29^ have found that a lack of confidence in bioequivalence tests directly implies the reluctance of some physicians to prescribe generic drugs, resulting in a lack of information for users and, consequently, low confidence in the effectiveness of these drugs. However, among those using generic drugs, most report being satisfied with the results^9,23^.

Purchasing preferences can be understood as related to the concept of acceptability, a dimension of access to medicines that expresses the balance between the characteristics of products and services and the expectations and needs of users^7^.

Hanson et al.^15^ provides an integrated view of health systems with five levels (individuals, families and communities, provision of health services, health sector, intersectoral policies, and international and regional level)^15^. This proposal entails the identification of the main barriers to access services and products at each level, as well as their interactions. The case of access to generic drugs is exemplary in this regard, since it involves aspects at the individual level, such as preferences and acceptability, and aspects outside the health sector, such as performance of the retail trade and advertisement of the producers of the drug.

This study aims to identify factors associated with the preference for purchasing generic drugs in a medium-sized municipality in Southern Brazil.

## METHODS

A population-based cross-sectional study was carried out in the municipality of Pelotas, in the State of Rio Grande do Sul, Brazil. The city has approximately 328,000 inhabitants, of which 93.2% live in urban areas. Its Human Development Index in 2010 (HDI 2010) was 0.739 (Population Census of 2010 of the Brazilian Institute of Geography and Statistics – IBGE). This study was part of the Research Consortium aiming to assess the health of adolescents, adults, and older adults in the city^6^. Data were collected from February to June 2012.

Sampling process was carried out in multiple stages. The primary sampling unit was 495 census tracts (delimited areas covering approximately 300 households each). Of these tracts, 130 were selected and stratified by socioeconomic status to ensure coverage of the entire city. The secondary sampling unit was the household. In each sampled tract, between 50 and 60 households were systematically selected, proportionate to the size of the tract, amounting to 1,722 households in the sample. In each selected household, all individuals aged 20 years or older were invited to participate, except those with severe mental disability that made it impossible to answer to the questionnaire.

Sample size was based on two different calculations: estimated prevalence of “preference for purchasing generic drugs” and study of association between “preference for purchasing generic drugs” and independent variables. Considering the final sample size (n = 2,925), we could estimate prevalence rates between 32% and 40%, with an error margin of ± 5 percentage points and a 95% confidence level. In addition, relative risks of 1.15 or great could be detected with 80% power and 95% confidence level.

A pre-tested and standardized questionnaire was used to assess the variables of interest. The main outcome variable was based on a single question: “When you go to pharmacy to purchase medicines, do you prefer to purchase generic drugs?”.

Independent variables were: a) demographic and socioeconomic: sex, age (20–39, 40–59, ≥ 60 years); skin color/race (white, black, yellow, brown, or indigenous), education (0–4, 5–8, ≥ 9 years of education); asset index (AI), based on the ownership of 13 assets (education of the household head, number of rooms used to sleep, number of bathrooms, colored television, car, radio, refrigerator or freezer, DVD, telephone line, desktop, air conditioner or split air conditioner, vacuum cleaner, and domestic housemaids; first – 20% poorest –, second, third, fourth, and fifth quintiles – 20% wealthiest)^5^; b) health: drugs used for chronic or eventual diseases; self-reported health status (excellent or very good, good or fair, poor); c) knowledge on generic drugs, using a score. This score, ranging from zero to three, included the answers to questions about the price of generic drugs as compared to the reference drug (the correct answer was “lower”), the quality of generic drugs as compared to the reference drug (the correct answer was “the same”), and the correct identification of at least one of the characteristics that differentiate the packaging of generic drugs from other drugs (Generic law number printed on the packaging, the letter “G” for generic drugs, or the words “generic drugs”). These questions composing the score were applied before the question about the outcome.

The ability to recognize the packaging of generic drugs was also tested. First, respondents were presented with the image of a reference drug (Voltaren^®^). Then, the image of the packaging of the similar drug was shown (Diclosódico) and we asked whether or not it was a generic drug (correct answer = no). After, the image of the packaging of the generic drug was presented (*diclofenaco de sódio* – *diclofenac sodium*) and we asked if the drug was generic (correct answer = yes). This test was applied after the question about the outcome. The ability to recognize the packaging of generic drugs was always assessed in the same order (reference drug, similar drug, and generic drug) and just once.

Analyses were conducted in Stata 12.1^b^, considering the design effect and using the set of *svy* commands specific to the analysis of surveys based on complex sampling strategies. Bivariate analyses were performed to calculate the prevalence of the outcome in each category of predictors, as well as unadjusted prevalence ratios and their 95% confidence intervals. For statistical significance, we used the p-values obtained by the Wald test for heterogeneity or linear trend.

**Figure 1:**
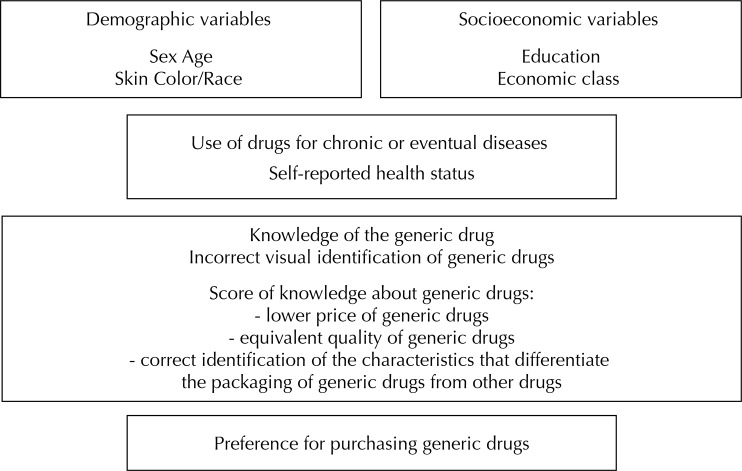
Hierarchical model of analysis on the determinants of “prevalence of preference for purchasing generic drugs”.

In the adjusted analyses, considering our dichotomous outcome with a high prevalence, a Poisson regression with robust adjustment for variance was used^4^. Analyses were based on a hierarchical model^34^ (Figure 1). Therefore, the effect of each predictor on the outcome was adjusted for all variables in its same level or above in the causal pathway. Demographic (sex, age) and socioeconomic variables (education, asset index) were placed at the distal level of determination. On the second level, we included self-reported health status and in the third level, the knowledge about generic drugs.

An informed consent was obtained from each subject and the research protocol was approved by the Ethics Committee of the Faculdade de Medicina da Universidade Federal de Pelotas.

## RESULTS

Within the sampled households, we located 3,379 eligible individuals, of which 2,925 (86.6%) answered the questionnaire. For this specific analysis, the sample size was 2,856 (84.5%), because some participants had missing values for the outcome variable. Losses and refusals had a higher proportion for males (56.6%), but they were similar to the sample in relation to average age.

The preference for purchasing generic drugs was 63.2% (95%CI 61.4–64.9). Table 1 describes the sample according to the demographic, socioeconomic, and health variables and the self-assessment score of knowledge about generic drugs. Most participants were female (59.2%), the mean age was 45.5 years (SD = 16.4), most were white (80.1%), more than half had nine or more years of study (54.7%) and a large fraction of the sample (64.4%) declared to be in good health.

**Table 1:** Description of adults according to the demographic and socioeconomic variables, health status, and knowledge about generic drugs. Pelotas, State of Rio Grande do Sul, Brazil, 2012.

Variable	n	%
Demographic and socioeconomic variables		
Sex		
Female	1,691	59.2
Male	1,165	40.8
Age (years)		
20–39	1,132	39.6
40–59	1,094	38.3
60 or more	630	22.1
Skin color/Race		
White	2,289	80.1
Black	345	12.1
Other^a^	222	7.8
Education (years of study)		
0–4	495	17.4
5–8	796	27.9
9 or more	1,562	54.7
Asset index (quintiles)^b^		
1st (20% poorest)	555	19.6
2nd	538	19.0
3rd	583	20.6
4th	588	20.7
5th (20% wealthiest)	570	20.1
Health status		
Use of drugs for chronic or eventual diseases		
Chronic	1,615	72.1
Eventual	625	27.9
Self-reported health status		
Excellent or very good	889	31.1
Good or fair	1,840	64.4
Poor	127	4.5
Knowledge about generic drugs		
Lower price of generic drugs		
No	339	11.9
Yes	2,517	88.1
Equivalent quality of generic drugs		
No	864	30.2
Yes	1,992	69.8
Identification of characteristics that differentiate the packaging of generic drugs		
Correct identification	2,189	76.6
Incorrect identification	667	23.4
Incorrect visual identification of similar medicines as generic drugs		
No	2,057	72.0
Yes	799	28.0
Score of knowledge about generic drugs^c^		
0	76	2.7
1	350	14.9
2	942	33.0
3	1,488	52.1

Total	2,856	100

^a^ Yellow, brown, or indigenous.

^b^ The maximum value of information lost was 22 missing (0.77%).

^c^ Score was composed of the answers to three questions: the price of generic drugs being lower, the quality of generic drugs being equivalent to the reference drug, and the correct identification of at least one of the characteristics that differentiated the packaging of generic drugs from other drugs.

Most participants were aware that generic drugs were cheaper than reference medicines (88.1%), had an equivalent quality (69.8%), and were able to correctly identify the characteristics that differentiate the packaging of generic drugs from other drugs (76.6%). The score created based on this knowledge showed that 52% of respondents achieved maximum value. In addition, 72% of the participants could correctly distinguish the packaging of a similar drug from that of a generic medicine (Table 1).

Table 2 shows the prevalence and factors associated with the preference for purchasing generic drugs. Men had a greater preference for purchasing generic drugs compared to women (PR = 1.08; 95%CI 1.03–1.14), and the effect remained significant after adjusting for age, education, and asset index.

**Table 2:** Associated factors with preference for purchasing generic drugs in adults of Pelotas, State of Rio Grande do Sul, Brazil, 2012. (n = 2,856)

Level^a^	Variable	n (%)	Crude PR^b^ (95%CI)	p^c^	Adjusted PR^d^ (95%CI)	p^c^
Demographic and socioeconomic variables						
1	Sex			0.004		0.003
	Female	703 (60.3)	1		1	
	Male	1,101 (65.1)	1.08 (1.02–1.14)		1.08 (1.03–1.14)	
1	Age (years)			< 0.001		0.021
	20–39	760 (67.1)	1.15 (1.06–1.24)		1.10 (1.02–1.20)	
	40–59	675 (61.7)	1.05 (0.96–1.15)		1.03 (0.94–1.12)	
	60 or more	369 (58.6)	1		1	
1	Skin color/Race			0.565		-
	White	1,436 (62.7)	1		-	
	Black	222 (64.4)	1.02 (0.93–1.13)		-	
	Other^e^	146 (65.8)	1.05 (0.95–1.15)		-	
1	Education (years)			0.005		0.008
	0–4	284 (57.4)	1		1	
	5–8	529 (66.5)	1.15 (1.06–1.26)		1.16 (1.05–1.27)	
	9 or more	988 (63.3)	1.10 (1.01–1.20)		1.13 (1.03–1.25)	
1	Asset index (quintiles)			0.058		0.033
	1st (20% poorest)	371 (66.9)	1.15 (1.03–1.27)		1.18 (1.06–1.33)	
	2nd	360 (66.9)	1.15 (1.03–1.28)		1.15 (1.02–1.30)	
	3rd	367 (63.0)	1.08 ( 0.96–1.21)		1.07 ( 0.95–1.21)	
	4th	365 (62.1)	1.06 (0.95–1.20)		1.06 (0.94–1.19)	
	5th (20% wealthiest)	332 (58.3)	1		1	

Health status						

2	Use of drugs for chronic or eventual diseases					
	Chronic	1,005 (63.8)	1	0.590		-
	Eventual	570 (36.2)	0.98 (0.91–1.05)		-	
2	Self-reported health status					
	Excellent/Very good	555 (62.4)	1	0.071	1	0.026
	Good/Fair	1,157 (62.9)	1.00 (0.95–1.07)		1.01 (0.95–1.08)	
	Poor	92 (72.4)	1.16 (1.02–1.32)		1.20 (1.05–1.38)	

Incorrect visual identification of similar medicines as generic drugs						

3	No	1,325 (64.4)	1.07 (1.00–1.16)	0.052	0.98 (0.92–1.05)	0.608
	Yes	479 (60.0)	1		1	

Score of knowledge about generic drugs^f^						

3	0	14 (18.4)	1	< 0.001^g^	1	< 0.001^g^
	1	120 (34.3)	1.86 (1.14–3.03)		2.01 (1.20–3.37)	
	2	514 (54.6)	2.96 (1.90–4.62)		3.27 (2.04–5.27)	
	3	1,156 (77.7)	4.22 (2.70–6.58)		4.66 (2.89–7.52)	

	Total	1,804 (63.2)				

^a^ Level according to the hierarchical model of analysis.

^b^ PR: Prevalence Ratio.

^c^ Chi-Square test.

^d^ Adjusted PR: adjusted for the same level as the variables and lower levels that had p ≤ 0.20.

^e^ Yellow, brown, or indigenous.

^f^ Score was composed of the answers to three questions: the price of generic drugs being lower, the quality of generic drugs being equivalent to the reference drug, and the correct identification of at least one of the characteristics that differentiated the packaging of generic drugs from other drugs.

^g^ Wald test for the linear trend and ordinal variables.

The preference for purchasing generic drugs was inversely associated with age, both in the crude (p < 0.001) and adjusted (p = 0.021) analyses. The preference for purchasing generic drugs was 57.4% among persons with up to four years of education, 66.5% between five to eight years of education, and 63.3% among those with nine or more years of education (p = 0.008). After adjustment, there was a trend of increasing preference for purchasing generic drugs as the asset index decreased (p = 0.033).

Self-reported health was associated with the preference for purchasing generic drugs (p = 0.026). The participants who reported their health as “poor” were more likely to prefer generic drugs (PR = 1.20; 95%CI 1.05–1.38). Skin color/race and the use of drugs for chronic or eventual diseases were not associated with the preference for purchasing generic drugs (Table 2).

Among those who had good knowledge about generic drugs, 77.7% had a preference for purchasing them, whereas this proportion was only 18.4% among those with bad knowledge (p < 0.001). After adjustment, there was a trend of increasing preference for purchasing generic drugs as the knowledge about them increased (p < 0.001). Participants who answered correctly the three questions about the drugs were more likely to prefer generic drugs (PR = 4.66; 95%CI 2.89–7.52) (Table 2).

**Figure 2:**
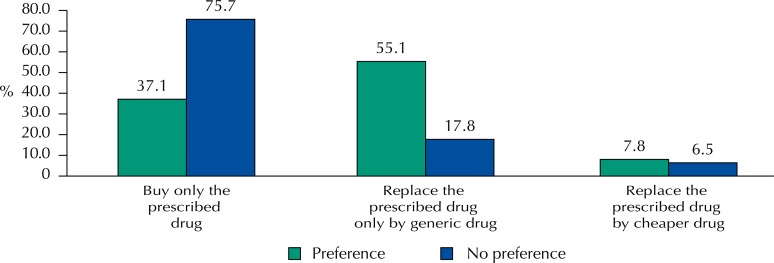
Strategies used to purchase drugs among those with preference and not preference for the generic drug. Pelotas, State of Rio Grande do Sul, Brazil, 2012. (n = 2,856)

Figure 2 shows the different strategies used to purchase drugs among those who prefer and do not prefer generic drugs. In general, those who did not prefer them tended to buy the medicine that was prescribed (75.7%) regardless of being a generic or not. However, 18% still ended up replacing the medicine prescribed by the generic drug. In addition, the main strategy used by those who preferred generic drugs was substitution (55.1%).

**Figure 3:**
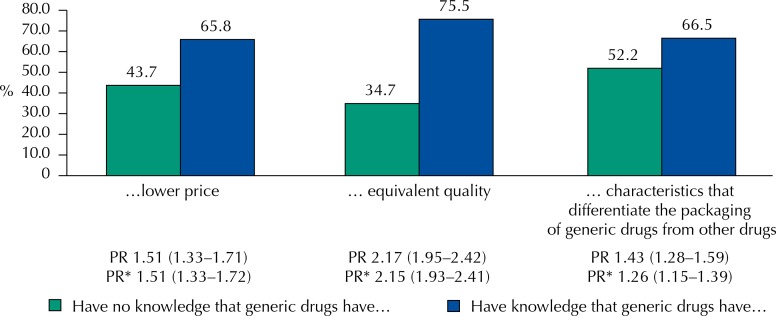
Preference for purchasing generic drugs according to knowledge about generic drugs. Pelotas, State of Rio Grande do Sul, Brazil, 2012.

Figure 3 shows the preference for purchasing generic drugs according to the knowledge of its characteristics. After controlling for demographic and socioeconomic variables, those who knew that the generic drug was cheaper than the reference one had a higher preference for purchasing generic drugs (51%; 95%CI 33–72) than those who were not aware of this difference in price. Those who had knowledge of the equivalent quality of generic drugs were 115% more likely to prefer generic drugs than those who did not know (95%CI 93–141). Those who correctly identified the characteristics that distinguish generic drugs from other medicines preferred the generic ones (26%; 95%CI 15–39).

PR: crude Prevalence Ratio

* Prevalence ratio adjusted for sex, age, education, and socioeconomic position (p < 0.001).

## DISCUSSION

The preference for purchasing generic drugs found in our study was similar to the one reported in another research conducted in Brazil, which has indicated that 68% of Brazilians preferred to use generic drugs^c^. A study performed in 2007 study found a preference of 60.7%^35^. These findings may suggest an increased acceptance of generic medicines in Brazil. However, it is known that the hypothetical preference or positive attitude towards generic drugs does not necessarily translate into consumption in itself^16^. In addition, users can prefer the generic drug, but in the moment of purchase they can choose the similar drug, because of other factors such as price, supply, or availability.

According to the hierarchical model of analysis, the proximal variables had more effect on the preference than distal variables (Figure 1). The main factors affecting the preference for purchasing generic drugs were the socioeconomic characteristics, especially those related to perception and knowledge about generic drugs. In this regard, data in the literature are contradictory^16,29,31^. Differences in contexts, health policies, and financial incentives may have contributed to these contradictory findings.

Some studies have found results similar to ours, with respect to age^1^ including a review by Hassali et al.^16^, covering data from 1970 to 2008 for middle- and high-income individuals. However, Shrank et al.^31^ has found that, in Columbia, USA, younger persons were those who were less likely to prefer generic drugs.

Regarding sex in our study, more men preferred generic drugs, whereas Alujer et al.^1^ and Shrank et al.^31^ have found no sex differences in the preference for generic drugs in their studies in Albaceta (Spain) and Columbia (USA), respectively. Differences regarding sex could be due to the largest proportion of losses among males, which could have skewed the results because of sampling bias.

In terms of education, our findings are similar to those reported by Hassali et al.^16^, who have found that those with less education had more negative attitudes towards generic drugs. Regarding the socioeconomic position, our findings differ from the literature. While in our study the wealthiest class had lower preference for purchasing generic drugs, in the studies by Hassali et al.^16^ and Shrank et al.^31^, richer participants showed greater preference for the use of generic drugs than poorer participants. The results of our study showed a positive association between knowledge of generic drugs and purchase preference. In the study of Quintal et al.^29^ carried out with medicine users and pharmacists, they have been observed that the lack of information received by the user, lack of prescription, and lack of confidence in generic medicines were the main reasons for the underuse of generic drugs.

By observing the specific knowledge about price, quality, or characteristics of the packaging, we can note that those with better knowledge about generic drugs have higher preference for purchasing them, being the perception on equivalent quality the main determinant factor in the choice of generic drugs, similar to the findings of other studies^13,32^. Keenum et al.^22^ have observed that most participants agreed that generic drugs were cheaper (98%), were as effective as the reference medicine (77%), and reported not caring about the replacement (80%); but only 45% said they preferred generic drugs over brand name drugs^22^. This may be due to some prior negative experiences or guidance received from the prescriber^36^. This is similar to our finding that 18% of those who do not prefer generic drugs end up replacing the prescribed drug by the equivalent generic drug. Factors related to the behavior of consumers, prices, or even the suggestions of clerks may be influencing this choice. A study conducted in Brazil has shown differences on the perceptions regarding generic drugs^28^. Nardi et al.^28^ have pointed out price, effectiveness, and safety as important factors that may contribute to the decision to purchase generic drugs.

Another important barrier to increased use of generic drugs is the belief that generic drugs are less effective^3^. Although most persons accept the replacement, many do not accept it because they “believe that it does not have the same effect” or “it differs from the reference drug in pharmacology aspects”^1^. Many users remain skeptical because of the lower price of generic drugs, associating it with lower quality^17^.

One limitation of our study was that the sample was representative of one medium-sized city in Southern Brazil, and not the entire country. Pelotas tends to present better economic indicators than the national mean^18,19^. This characteristic limits the extrapolation of our findings to the entire country, but the data presented here are appropriate to point out directions for campaigns aiming at the expansion of the acceptance and use of generic medicine. We were able to indicate the main factors affecting the acceptance of generic drugs, which are the perception of users and the knowledge on the quality of these products.

Although we found great influence from the knowledge of those who prefer generic drugs, in cross-sectional studies, some results are difficult to explain because of reverse causality. However, evidence from our study and from the literature has shown that preference is influenced by knowledge^14,32^.

Additionally, we could not assess other aspects that may influence the preference for purchasing generic drugs, such as: user perception regarding the safety, efficacy, and effectiveness of generic drugs; previous experiences; preference of prescribers and their influence on the user; financial support; variables related to the use of health system; drug dispensing; and acceptance and knowledge of physicians about generic drugs. In this study, the preference for purchasing generic drugs was limited to individual factors. Studies have shown that prescribers have an important role in influencing the preference of users for generic drugs^1,10^. Despite having a more negative opinion than pharmacists about generic drugs^10^, the number of physicians who prescribe generic drugs has increased in Brazil in recent years^9,33^.

Another limitation of our study was the possible induced positive answer for the outcome because of the order that questions were applied. We reduced this limitation using a direct question to measure the outcome.

In this study, we assumed knowledge as being aware of the technical principles that generic drugs are cheaper and equivalent in terms of quality as compared to reference medicines. However, there are exceptions; for instance, a person may have experienced more expensive generic drugs than the reference product, because of market competition strategies. In addition, a product will hardly be effective in all cases. When the ineffectiveness is experienced with a generic, this negative experience can reinforce the belief of a lower quality. Thus, we find it appropriate to combine aspects of perception and knowledge to express both the technical aspects (price and quality) of generic drugs and their translation in the experience of users, which can affect their perception from personal experience or from persons around them.

In conclusion, those who showed lower preference for generic drugs were women, older adults, users with lower education, and those with a high asset index. We also observed that the greater the knowledge or positive perception about generic drugs, the greater the preference to purchase this product. Thus, educational campaigns for healthcare professionals and consumers seem to be the best strategy to expand the use of such products, especially with regard to clarifications on quality. This needs to be paired with policies to ensure the supply of generic drugs at the cheapest prices among all medicines available in the market.

## Footnotes

a Associação Brasileira das Indústrias de Medicamentos Genéricos. Mercado dos medicamentos genéricos. São Paulo; 2015.

b StataCorp. Stata Statistical Software: Release 12. College Station, TX: StataCorp LP; 2011.

c Instituto de Ciência, Tecnoilogia e Qualidade, Departamento de Pesquisa. Hábitos de consumo de medicamentos. São Paulo: ICQT; 2013.
